# Controlling Pre-leukemic Thymocyte Self-Renewal

**DOI:** 10.1371/journal.pgen.1004881

**Published:** 2014-12-18

**Authors:** Steven Goossens, Pieter Van Vlierberghe

**Affiliations:** 1 VIB Inflammation Research Center, Ghent University, Ghent, Belgium; 2 Department for Biomedical Molecular Biology, Ghent University, Ghent, Belgium; 3 Mammalian Functional Genetics Laboratory, Division of Blood Cancers, Australian Centre for Blood Diseases, Monash University, Melbourne, Victoria, Australia; 4 Center for Medical Genetics, Ghent University Hospital, Ghent, Belgium; Cincinnati Children′s Hospital Medical Center, United States of America

T cell acute lymphoblastic leukemia (T-ALL) develops in a multistep process whereby thymic progenitor cells gradually accumulate genetic and epigenetic changes, eventually leading to fully transformed immature lymphoblasts. Aberrant activation of transcription factor oncogenes is considered a core component of the oncogenic program that drives malignant T cell transformation. For example, the TAL1 (SCL) and LYL1 basic Helix-Loop-Helix (bHLH) transcription factors, as well as the LIM domain–only proteins LMO1 or LMO2, are activated by chromosomal translocations or interstitial deletions in a large fraction of primary T-ALLs. Notably, these leukemias often present with activating *NOTCH1* mutations, suggesting that enhanced *NOTCH* signaling and aberrant *TAL1*, *LYL1, LMO1*, and/or *LMO2* expression are collaborative events in the multistep pathogenesis of T-ALL [1], [2]. Recent experimental evidence uncovered the existence of long-lived pre-leukemic stem cells (pre-LSCs) with self-renewal properties, allowing clonal expansion and subsequent acquisition of oncogenic mutations leading to cancer [3], [4]. For example, *DNMT3A* mutant pre-LSCs were shown to survive chemotherapy and represent a reservoir for leukemic progression and hematological relapse in acute myeloid leukemia (AML) [5], [6]. Although the concept of pre-leukemic stem cells has been previously proposed in the context of T-ALL [7], the actual molecular mechanisms by which T cell-specific oncogenes regulate pre-LSC activity of thymic precursors remains largely unexplored. Notably, these mechanistic insights could provide valuable input for the development of novel therapeutic strategies that can effectively eradicate quiescent and therapy-resistant clones [4], [6], [7].

## Pre-leukemic Stem Cells: It's All in the DN3

**Figure 1:**
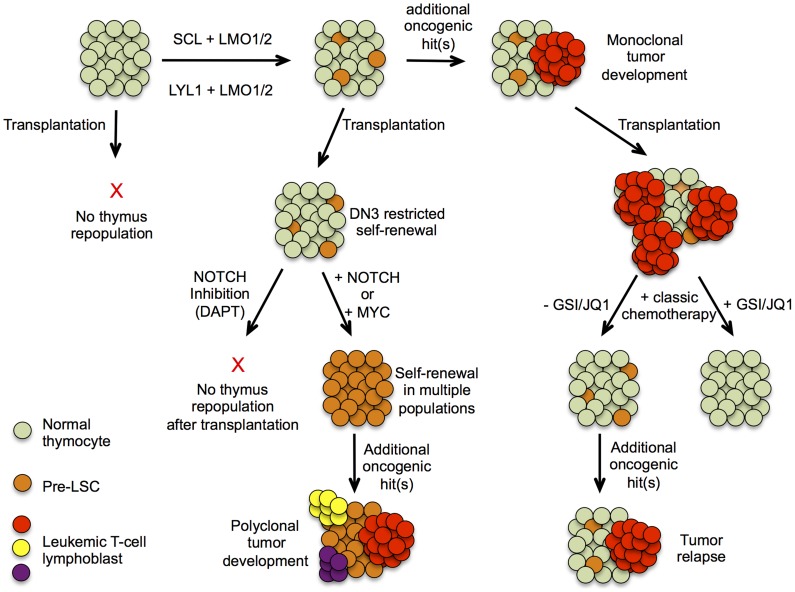
Hypothetical model describing pre-leukemic stem cells formation and T-ALL development in *SCL^tg^LMO1^tg^* mice. Schematic presentation of T-ALL progression based on experimental evidence presented by Gerby et al. Upper part represents the progression of T-ALL development in a thymus initiated by the formation of a pre-leukemic stem cell population, allowing clonal expansion and subsequent acquisition of oncogenic mutations, eventually leading to a mono/oligoclonal tumor. The lower part depicts results obtained after transplantation of thymocytes at different stages of tumor development and the effects of altering NOTCH1 signaling or its downstream component MYC on their repopulating capacity.

In this issue of *PLOS Genetics*, Gerby et al. [8] used the *SCL^tg^LMO1^tg^* transgenic mouse model [9] to investigate the molecular pathways that mediate the transition of normal thymic precursors into pre-LSCs. Interestingly, combined *SCL* and *LMO1* overexpression was sufficient to reprogram double negative 3 (DN3) T cell precursors with a finite lifespan into pre-LSCs with self-renewal activity and retained differentiation capacity (Fig. 1). Moreover, transcriptome analysis revealed that this transition towards pre-LSC was accompanied by a marked up-regulation of a stem cell signature, including Hhex, Nfe2, and Lyl1. Notably, Gerby et al. [8] also used *LYL1^tg^LMO1^tg^* transgenic mice to show that combined *LYL1* and *LMO1* overexpression results in similar reprogramming activities, as observed for SCL-LMO1. These results confirm recent work from McCormack and colleagues showing Lyl1 as an essential, but not sufficient, factor to reprogram *Lmo2* overexpressing DN3 cells into pre-LSCs [10]. All together, these data convincingly show that combined activation of SCL or LYL1 together with LMO1 or LMO2 can drive oncogenic reprogramming of DN3 thymic precursors into self-renewing pre-LSCs.

## NOTCHing up Pre-leukemic Stem Cells

High physiological levels of Notch1 signaling drive irreversible T-lineage commitment at the DN3 stage of T cell differentiation. Therefore, enhanced Notch1 activity could be critically involved in the reprogramming capacity of SCL-LMO1. Strikingly, Gerby and colleagues [8] showed that the self-renewal capacity of *SCL^tg^LMO1^tg^* DN3 cells was completely abrogated upon ablation of Notch1 activity by γ-secretase inhibitor treatment (Fig. 1). For the first time, these results unambiguously show that high physiological levels of Notch1 are critically required for SCL-LMO1–driven reprogramming of DN3 thymocytes into self-renewing pre-LSCs. Conversely, Gerby et al. [8] showed that supraphysiological levels of Notch1 significantly expanded the pool of pre-LSC; enhanced their engraftment and rendered them independent of the thymic microenvironment (Fig. 1). Remarkably, Notch1 activation by itself was not sufficient to trigger reprogramming activity, suggesting that the Notch1 oncogene is devoid of intrinsic self-renewal capacity, but confers a proliferative advantage to SCL-LMO1–primed pre-LSCs (Fig. 1). If the molecular mechanisms, as described above, turn out to be conserved in the human setting, inhibition of NOTCH signaling by γ-secretase inhibitors, small molecule inhibitors or antibodies targeting specific Notch receptors, could serve as an attractive approach to effectively target pre-LSCs for the treatment of human T-ALL. Most notably, in that scenario, eradication of the quiescent and chemo-resistant pre-LSCs by inactivation of NOTCH signaling might be applicable for a large fraction of primary human T-ALL patients, irrespective of their NOTCH1 mutational status at diagnosis. Furthermore, Gerby et al. [8] also showed that Myc acts as a critical mediator of pre-LSC activity downstream of Notch1, suggesting that combined NOTCH/MYC inhibition by γ-secretase and BET-bromodomain inhibitors [11] could further potentiate therapeutic targeting of the pre-LSC population in the context of human T-ALL (Fig. 1).

## Thymocyte Self-Renewal: In Which E Proteins Meet LMOs

From a mechanistic point of view, it has been previously postulated that the oncogenic potential of SCL and LYL1 is mediated by inhibition of E proteins [12]. Indeed, SCL and LYL1 can heterodimerise with E2A or HEB and form inactive transcriptional complexes that drive repression of E protein target genes, including critical regulators of T cell differentiation (Ptcra, Il7r, and Rag2) [12]. Alternatively, SCL and LYL1 can directly interact with LMO1 or LMO2 and recruit other co-activators to form active multiprotein transcriptional complexes that drive specific target gene expression [13]. Up until now, no concrete proof existed for whether the sequestration of E proteins is truly the major cause of SCL-driven leukemia development. Here, the authors generated a transgenic mouse model expressing a mutant form of SCL (SCLm13) that is defective in LMO1/2 binding, but still inhibits E proteins through heterodimerization [8]. Strikingly, the authors observed a strong delay in leukemia onset and a reduced tumor penetrance in the *SCLm13^tg^LMO1^tg^* versus *SCL^tg^LMO1^tg^* transgenic mice, suggesting that E protein inhibition is not the main oncogenic property of SCL in this model of murine T cell leukemogenesis. Furthermore, combined *SCLm13* and *LMO1* overexpression was unable to reprogram DN3 cells into pre-LSCs. All together, these data suggest that the SCL-LMO1 interaction is essential for the generation of pre-LSCs through reactivation of stem cell genes (Hhex, Lyl1) and serves as a critical mediator of malignant T cell transformation. Given this, targeting the SCL-LMO1/2 or LYL1-LMO1/2 [10] protein–protein interaction by small molecule inhibitors might represent an elegant new therapeutic approach to constrain self-renewing capacity of both leukemic and pre-leukemic cancer stem cells in T-ALL.

In conclusion, this study [8] nicely illustrates how cooperative transcription factor oncogenes can reprogram normal T cells into pre-LSCs during the earliest steps of malignant T cell transformation. Although these results provide novel insights in the multistep pathogenesis of T-ALL and have important therapeutic implications, it remains to be established whether gain of pre-leukemic self-renewal capacity is a true obligatory trait for human T-ALL development. Moreover, it would be interesting to know if other T-ALL–specific transcription factor oncogenes, including *TLX1*, *TLX3*, or *HOXA*, also possess the intrinsic capacity to induce self-renewal in T cell progenitors [7]. In regard to this notion, it is currently unclear if the results presented in this study are broadly relevant for the majority of primary human T-ALLs or if they specifically apply to a particular molecular genetic subtype of this disease. For example, early immature T-ALLs show high expression levels of *SCL*, *LYL1*, *LMO1*, and *LMO2* as a reflection of their maturation arrest at the early double negative stages of T cell differentiation [14]. In contrast, chromosomal translocations or small genomic deletions targeting *SCL*, *LYL1*, and/or *LMO2*, are exclusively present in primary human T-ALLs with a late cortical tumor phenotype [1]. Therefore, the functional role of SCL, LYL1, and/or LMO2 in regulating self-renewal capacity might be influenced by the immunophenotypic background of the different molecular-genetic subtypes of human T-ALL, a notion that could be tested using transgenic models that overexpress the specific members of this bHLH transcription complex at later stages during T cell development.
